# Effects of Dehydration on Archery Performance, Subjective Feelings and Heart Rate during a Competition Simulation

**DOI:** 10.3390/jfmk5030067

**Published:** 2020-08-27

**Authors:** Alexandros Savvides, Christoforos D. Giannaki, Angelos Vlahoyiannis, Pinelopi S. Stavrinou, George Aphamis

**Affiliations:** Department of Life and Health Sciences, University of Nicosia, 46 Makedonitisas Avenue, CY1700 Nicosia, Cyprus; Savvides.A4@live.unic.ac.cy (A.S.); giannaki.c@unic.ac.cy (C.D.G.); vlahoyiannis.a@unic.ac.cy (A.V.); stavrinou.p@unic.ac.cy (P.S.S.)

**Keywords:** hydration, urine specific gravity, athletes, heart rate, fatigue, alertness

## Abstract

This study aimed to investigate the effect of dehydration on archery performance, subjective feelings and heart rate response. Ten national level archers performed two archery competition simulations, once under euhydration (EUH) and once in a dehydrated state (DEH), induced by 24-h reduced fluid intake. Hydration status was verified prior to each trial by urine specific gravity (USG ≥ 1.025). Archery score was measured according to official archery regulations. Subjective feelings of thirst, fatigue and concentration were recorded on a visual analogue scale. Heart rate was continuously monitored during the trials. Archery performance was similar between trials (*p* = 0.155). During DEH trial (USG 1.032 ± 0.005), the athletes felt thirstier (*p* < 0.001), more fatigued (*p* = 0.041) and less able to concentrate (*p* = 0.016) compared with the EUH trial (USG 1.015 ± 0.004). Heart rate during DEH at baseline (85 ± 5 b∙min^−1^) was higher (*p* = 0.021) compared with EUH (78 ± 6 b∙min^−1^) and remained significantly higher during the latter stages of the DEH compared to EUH trial. In conclusion, archery performance over 72 arrows was not affected by dehydration, despite the induced psychological and physiological strain, revealed from decreased feeling of concentration, increased sensation of fatigue and increased heart rate during the DEH trial.

## 1. Introduction

Hydration is of considerable interest for health, thermoregulation, as well as sports and athletic performance. A cascade of physiological responses even to a mild deficit in total body water hinders the body’s ability to thermoregulate and maintain blood flow [1]. Decreased plasma volume leads to decreased blood flow to the exercising muscles and contributes further to impaired aerobic capacity [1,2] on various athletic disciplines such as cycling [2,3], long-distance running [4] and rowing [5]. Water losses and ensuing hyperthermia during exercise can also impair team sports or tennis performance [6] and muscular endurance [7] but not maximum strength or power [8].

Archery requires a repetitive motion with great precision, and therefore, its physiological demands are not similar to other predominately aerobic or anaerobic sports. During each arrow throw, one arm is used to hold (push) the bow in a steady position while the other arm pulls the bow string, with increasing muscle tremor in order to hold arrow-target alignment until the release of the arrow [9]. It has been estimated that the average force required to pull the bow is 20 kg for the men and 18 kg for the women. When this is multiplied by the number of arrow throws during a competition, one can get the total workload required by the exercising muscles in the shoulder area [10] and the trapezoid muscle [11]. This requires potentially a degree of muscular endurance over the duration of the competition, which could be affected by dehydration [7], especially under certain environmental conditions, as for example increased ambient temperature. Furthermore, archers must maintain a steady posture and body alignment with the target, in order to be as successful as possible. Even moderate intensity exercise combined with dehydration can lead to altered posture and increased muscle tremor, whereas euhydration allows for good muscle function and retention of postural control [12].

In archers, cardiovascular system also undergoes a specific stress during training [13] and competition [14]. Quality shooting for hours has been shown to challenge cardiovascular fitness and hand-eye coordination [15]. Increased ambient temperature could amplify the burden of inadequate hydration levels, which in turn may lead not only to reduced aerobic performance [1], but also to decreased brain volume [16] and altered brain function [17]. Dehydration can also negatively affect mood and vigilance [18,19], and increase tension, anxiety and fatigue [18], adding to the existing archers’ competition stress.

So far, with regards to archery performance during competition, little is known about the effects of fluid restriction and dehydration on skill performance, cognitive function and especially hand-eye coordination, as well as subjective feelings of fatigue and concentration of archers. Notably, findings from a laboratory setting may differ from actual results in a real competition scenario, and thus, the primary aim of the present study was to investigate archery performance under a dehydration state, following 24 h of restricted fluid intake, during a simulated competition in a hot environment. Secondary aims were to monitor heart rate (HR) responses and subjective feelings related to dehydration (thirst, alertness, concentration, fatigue) during an archery competition simulation.

## 2. Materials and Methods

### 2.1. Participants

Ten national level archers (males *n* = 7, females *n* = 3; age: 22 ± 3 year, height 171 ± 9 cm, body mass 74.4 ± 11.9 kg) volunteered for this study. All participants were at the advanced level, training regularly 5–6 days per week, 2–3 h per day and competing for at least two years in Division A of the national championship. Any kind of history of major disease or medication was strictly considered as exclusion criterion. All athletes were informed for the purposes of the study and provided a written consent form. The study was approved by the Cyprus National Biothetics Committee on 5th July 2019 (Project ID: EEBK/EΠ/2018/13). All procedures were conducted according to the manual of the Declaration of Helsinki in 1964 and its later amendments.

### 2.2. Study Design

**Figure 1:**
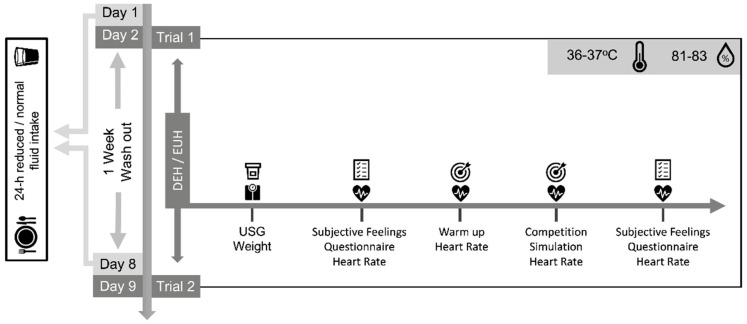
Overview of study design. DEH: Dehydration; EUH: Euhydration; USG: Urine Specific Gravity.

An overview of study design is depicted in Figure 1. Data collection took place during the competitive period at the Cyprus Archery Federation facilities. During this study, the temperature was 36−37 °C, and relative humidity was 81–83%. Participants visited the accredited archery area on two occasions, in counterbalanced order, once under euhydration (EUH) and once under a dehydrated state (DEH) induced by a 24-h controlled fluid intake. The two trials took place in the morning (warm up started at 8:20 a.m. and the last arrow throw was completed by 10:50 a.m.) 7 days apart. Upon arrival at the archery area, anthropometrics were measured and hydration levels were assessed. Participants underwent a warm-up, followed by the competition simulation. Heart rate was monitored through the trials. Self-perceived feelings were recorded at the start and end of each session.

### 2.3. Fluid Restriction Protocol

All participants received dietary instructions to follow during all the study protocol, in order to avoid any nutrient deficiency. Caffeinated beverages, nutritional supplements and alcohol were not permitted for 48 h prior to trials. During the last 24 h prior to the trials, the participants followed the same isoenergetic nutritional plan, with a macronutrient distribution of 20%, 55% and 25% for protein, carbohydrate and fat, respectively. The nutrition plan was analyzed with Nutrilog Software v 2.60 (Marans, France), and the water content of the diet was approximately 1.2 L (0.9 L as water content of foods and 0.3 L as a result of macronutrient oxidation). In the dehydration scenario, the archers were further provided with 250 mL fluids at five intervals (total amount of fluids 5 × 250 mL = 1.25 L) during the last 24 h before the competition simulation. In the euhydration scenario, fluid intake was also standardized, providing archers with 500 mL fluids at breakfast, lunch and dinner and 250 mL of fluids at six intervals across the day (total amount of fluids (3 × 500 mL) + (6 × 250 mL) = 3 L).

### 2.4. Archery Competition Simulation Protocol

The competition simulation protocol consisted of a standardized warm-up, followed by the main archery competition phase. During the warm-up, each participant occupied one shooting line and upon a signal from the referee the participants shot 2 sets of 6 arrows at a 5-m distance targets over a 4-min period with a 3-min break between sets in order to collect the arrows. Then, the participants shot 2 rounds of 6 arrows at 70 m targets, again each round within a 4-min time frame, with 3 min break for arrow collection, and rested for 10 min before the main competition phase began.

During the competition phase, the archers assumed their position in their respective shooting lines and shot 6 rounds of 6 arrows. According to the regulations, 4 min were allowed for each round to be completed, followed by a 3-min break to collect arrows and write down the score for each participant. This was followed by a 10-min rest break, before continuing with another round of 6 arrows, until all 6 rounds were completed. The archery competition simulation was conducted according to the regulations of the International Archery Federation, at the Olympic distance of 70 m. All health and safety measures were taken according to the international competition rules. No food or fluid ingestion was allowed during the two main trials.

### 2.5. Anthropometrics

Upon arrival at the archery area, body mass (kg) was measured (participants wearing shorts and t-shirt only) using a portable scale (Seca model 755, Hamburg, Germany). Height (cm) was measured with a standing stadiometer (Seca model 720, Hamburg Germany).

### 2.6. Hydration Status Assessment

In the morning of each experimental trial, before study procedures, participants provided a first-morning-urine sample in a 60-mL container. Urine specific gravity (USG) was measured upon arrival of the participants at the archery center using a urine refractometer (DIGIT 0−12, Medline Scientific Limited, UK), to record hydration status. Euhydration/hypohydration cut-off point was set at USG < 1.025 [20]. All remaining urine was disposed down the toilet immediately after. No biological samples were stored after the determination of urine specific gravity.

### 2.7. Heart Rate Response

After urine collection, participants were allowed to rest, sitting comfortably, while a heart rate monitor (Polar H7, Polar Electro Oy, Professorintie S, FI-90440, Kempele, Finland) was attached on the participants’ chest in order to continuously monitor heart rate throughout the exercise trials. Heart rate during the trials was recorded at baseline, end of the rest break after throw 6 and at the end of each throw.

### 2.8. Subjective Feelings Monitoring

At baseline and at the end of each trial, participants completed a subjective feelings questionnaire [21]. Athletes self-rated their feelings on thirst, fatigue, alertness and ability to concentrate on a 0–10 visual analogue scale, where “0” was “not-at-all” and “10” was “very much”.

### 2.9. Statistical Analysis

Statistical analysis was performed with IBM^®^SPSS^®^ statistics for Windows, version 25.0 (IBM Corp, Armonk, NY, USA). Data are reported as mean ± standard deviation. Differences on archery performance (total score) were detected with a paired-samples *t*-test. The normality of data was assessed by the Kolmogorov–Smirnov test. All data were normally distributed, and comparisons on subjective feelings between trials and over time (baseline vs. end of archery competition) were made with a two-way Repeated Measures ANOVA (time point × trial). Heart rate response at rest and after arrow throw was also analyzed with two-way Repeated Measures ANOVA (time point × trial). Bonferroni post-hoc analysis was performed where necessary. Effect sizes were calculated using partial eta squared (η^2^) interpreted as 0.01 for small, 0.06 for moderate and 0.14 for large. Statistical significance was set at *p* < 0.05. Statistical power analysis was performed using the G*Power 3.1 power analysis software. Post hoc power analysis revealed that the sample size of the present study was adequate to provide statistical power of both heart rate and other main parameters of the study such as fatigue, concentration and thirst) with >90% power and with a significance level, α = 0.05.

## 3. Results

Measurements of USG at baseline showed that USG at the DEH trial was 1.032 ± 0.005, which was above the dehydration cut off level for all athletes, and it was also higher (*p* < 0.001) than USG at the EUH trial (1.015 ± 0.004). This confirms that the participants performed the archery competition under the desired hydration state at each trial.

**Figure 2:**
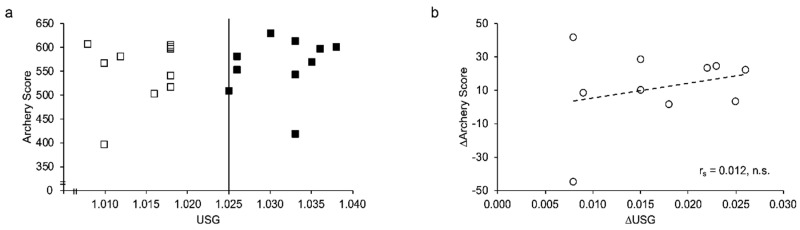
Archery Performance in relation to Urine Specific Gravity (USG) values. Panel (**a**) depicts both archery scores for EUH trial (open squares) and DEH trial (dark squares). Panel (**b**) depicts the correlation between ∆USG (USG_DEH_-USG_EUH_) and ∆Archery score (Archery Score_DEH_–Archery Score_EUH_). No significant correlation was found between USG and Archery Performance.

Total archery score was not different between trials (EUH 550 ± 63 points vs. DEH 562 ± 59 points; (*p* = 0.155)). No significant correlation was found between archery performance and USG levels (Figure 2).

**Figure 3:**
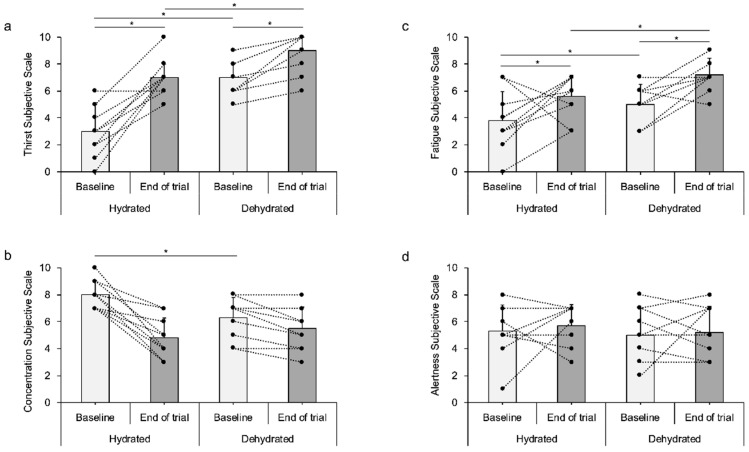
Subjective feelings of (**a**) Thirst, (**b**) Concentration, (**c**) Fatigue and (**d**) Alertness, at baseline and at the end of both Hydration (EUH) and Dehydration (DEH) trials. * Denotes statistically significant differences at the 0.05 level (2-tailed).

Subjective feelings analysis (Figure 3) showed different responses between conditions. Regarding thirst, there was a trial effect (F_1,9_ = 45.6, *p* < 0.001, η^2^ = 0.836), a time effect (F_1,9_ = 56.5, *p* < 0.001, η^2^ = 0.863) and a time × time interaction (F_1,9_ = 10.5, *p* = 0.010, η^2^ = 0.538), as sensation of thirst was higher at baseline of the DEH trial compared with EUH (*p* = 0.003) and thirst increased over time during both trials (EUH *p* < 0.001; DEH *p* < 0.001), but the magnitude of change of thirst during the trial was different between conditions. Regarding fatigue, there was a trial effect (F_1,9_ = 5.7, *p* = 0.041, η^2^ = 0.388) and a time effect (F_1,9_ = 21.8, *p* = 0.001, η^2^ = 0.708). The sensation of fatigue was higher at baseline of the DEH trial compared with EUH (*p* = 0.016), and fatigue increased over time in both trials (EUH *p* = 0.050; DEH *p* = 0.003). Analysis on concentration did not show any trial effect (F_1,9_ = 1.6, *p* = 0.244, η^2^ = 0.147), but there was a time effect (F_1,9_ = 32.7, *p* < 0.001, η^2^ = 0.784) and a time × trial effect (F_1,9_ = 28.2, *p* < 0.001, η^2^ = 0.758). Concentration scores were lower at baseline of the DEH trial compared with the EUH trial (*p* = 0.009), but by the end of the trial, concentration feeling was similar between trials (*p* = 0.320) as this feeling was stable during the DEH trial (*p* = 0.260) and decreased in the EUH trial (*p* < 0.001). Analysis of alertness showed that there was no trial (F_1,9_ = 1.7, *p* = 0.223, η^2^ = 0.160), no time effect (F_1,9_ = 0.20, *p* = 0.660, η^2^ = 0.023), and neither a time × trial interaction (F_1,9_ = 0.18, *p* = 0.678, η^2^ = 0.020).

**Figure 4:**
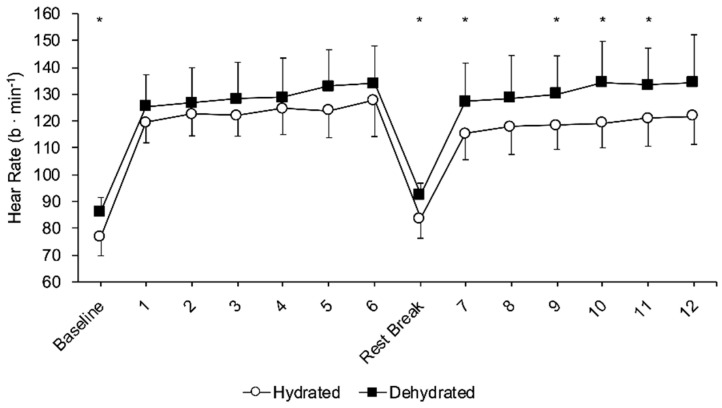
Heart rate response to hydration (open circles) and dehydration (dark squares) state, during the archery competition simulation. * Indicates difference between trials (*p* < 0.050). During exercise, heart rate was higher than baseline in both trials.

Heart rate response analysis (Figure 4) indicated a trial effect (F_1,9_ = 9.9, *p* = 0.012, η^2^ = 0.523), and a time effect (F_13,117_ = 136.3, *p* < 0.001, η^2^ = 0.931) as HR during the trial was above baseline in both conditions, and there was also a time × trial effect (F_13,117_ = 2.2, *p* = 0.006, η^2^ = 0.199). Post hoc analysis showed that HR at baseline was higher in the DEH trial (*p* = 0.003), but it was similar during the first six throws (*p* > 0.106). During the short resting period after throw 6, HR remained elevated in the DEH trial compared with EUH trial (*p* = 0.005), and HR was significantly higher during the DEH trial at throws 7 (*p* = 0.041), 9 (*p* = 0.043), 10 (*p* = 0.016) and 11 (*p* = 0.034). The statistical difference between the two trials at throw 8 was *p* = 0.086 and at throw 12 *p* = 0.075, indicating a trend towards higher HR during the DEH trial.

## 4. Discussion

In the current study, it was shown that dehydration induced psychological and physiological strain but did not alter shooting performance of national level archers during a competition simulation in hot environment. Specifically, the results of the current study indicate that archery score was not affected by dehydration. Nevertheless, significant differences on HR, subjective feelings of fatigue, and concentration were observed between conditions.

A compelling amount of evidence suggests that overall fitness, core strength, handgrip, upper body strength and static balance are related to archery performance and high scores [22,23]. In the present study, archery performance was similar between dehydration and euhydration trials; thereby, any potential effect on muscle function was not observed. Previous studies demonstrated that dehydration may decrease upper body muscle power during a maximum intensity anaerobic test [24] and during a fatiguing isometric strength protocol of repeated efforts at 85% of maximum voluntary contraction [7]. Nevertheless, the stress on the muscles during those tests was much higher than the effort during archery, and thus, it could be assumed that this is the reason why the participants in the present study were similarly successful during the two trials.

It has been proposed that the performance of experienced archers lies beyond their strength and fitness levels, to the mental domain, as the ability to concentrate is of utmost importance [25]. Dehydration may lead to impaired attention and motor coordination [26], decreased concentration [21,27,28] and increased sense of fatigue [21,28,29]. Additionally, dehydration has been linked to decreased sport-specific performance, such as decreased ball throwing accuracy in cricket [30] and basketball throw accuracy [31]. Mental fatigue has been shown to impair tennis serve and ground strokes accuracy [32]. The effects of dehydration may be more intense in hot and humid conditions due to a potentially increased thermal strain, which can affect the nervous system, cerebral blood flow and increase mental fatigue [33]. In this investigation, participants reported that they felt less able to concentrate and more fatigued at the dehydration baseline. Despite these relatively negative feelings, the overall archery score was not affected, even though the trials took place under conditions of high ambient temperature and humidity. Literature shows that experienced archers have higher emotional intelligence and emotional regulation; thereby, under stressful conditions, they may respond with higher consistency in cognitive processes during shooting [34].

Except for the induced psychological burden, dehydration had a significant physiological effect on the participants cardiovascular system. During the dehydration trial, heart rate was higher at baseline and during the second half of the competition simulation. In response to dehydration, heart rate may rise in order to maintain blood pressure and oxygen delivery [35]. A higher heart rate has been shown to increase tremors [36], which can potentially affect shooting performance. Indeed, higher heart rate has been associated with decreased performance in pistol shooting [37] and archery performance [14,38], since target aiming requires great stability by the archers and a high level of hand-eye coordination. Although archery performance was not affected over 72 arrow throws in the present study, during an official competition this volume of arrow throws equates to half of the anticipated number of throws until a winner is decided. Thus, as heart rate increased significantly during the latter stages of this investigation, it is unknown whether the magnitude of these changes in heart rate and aiming precision would be greater during a longer official competition with 140 arrow throws. Therefore, archers are advised to follow the current recommendations for fluid intake during exercise according to ACSM guidelines [39].

To the best of our knowledge, no study to date explored the effect of dehydration on archery performance in the field. Although it falls beyond the scope of the present study, it could be interesting to investigate the mediating effect of hormonal responses during this dehydration scenario into a larger sample size. Furthermore, as self-perceived psychological burden may compromise performance, a long-term observational investigation of hydration status and performance in archers would be of great value and could possibly elucidate physiological adaptations, underlying the current results. In addition, future studies on archery could include examination of secondary variables such as grip strength, or cognitive tests, in order to gain knowledge on specific physiological mechanisms or sports performance related parameters which can be affected by dehydration.

Limitations of the study. Potential limitations of the present study include the small sample size and absence of blood dehydration markers or body mass changes during the trials. Although urine specific gravity might not be the gold standard, the values of USG > 1.030 recorded in the present study, following fluid restriction, are a clear indication that dehydration was above 2% body mass [20], the critical dehydration point for athletic performance. Additionally, fluid restriction cannot be blunted in a real-world scenario. However, the strength of the present study lies on the fact that this is probably the first study in literature to directly address potential dehydration effects on advanced archers in a simulated competition, following controlled dehydration via fluid restriction.

## 5. Conclusions

To conclude, dehydration induced psychological and physiological strain in national level archers, revealed by decreased feeling of concentration, increased sensation of fatigue and increased heart rate during a competition simulation in a hot environment. The degree of this added burden did not affect archery performance over 72 arrow throws. Further investigation is required to elucidate the effect of the observed increased heart rate over time during the dehydration trial, which could potentially have a negative impact on aiming precision and archery performance over a complete competition with 140 arrow throws.
